# Health-Related Quality of Life in the Gender, Race, And Clinical Experience Trial

**DOI:** 10.1155/2011/349165

**Published:** 2011-08-28

**Authors:** Judith Feinberg, Michael Saag, Kathleen Squires, Judith Currier, Robert Ryan, Bruce Coate, Joseph Mrus

**Affiliations:** ^1^Department of Internal Medicine, University of Cincinnati College of Medicine, 231 Albert Sabin Way, Cincinnati, OH 45267, USA; ^2^Division of Infectious Disease, Department of Medicine, University of Alabama at Birmingham, Birmingham, AL 35294, USA; ^3^Division of Infectious Diseases and Environmental Medicine, Jefferson Medical College of Thomas Jefferson University, Philadelphia, PA 19107, USA; ^4^David Geffen School of Medicine, University of California, Los Angeles, Los Angeles, CA 90035, USA; ^5^Medical Affairs Biometrics, Tibotec Inc., Titusville, NJ 08560, USA; ^6^Clinical Affairs, Tibotec Therapeutics, Titusville, NJ 08560, USA

## Abstract

*Background*. We report health-related QoL (HRQoL) from GRACE (Gender, Race, And Clinical Experience) study by sex and race over 48 weeks. *Methods*. 429 treatment-experienced adults (HIV-1 RNA ≥ 1000 copies/mL) received darunavir/ritonavir 600/100 mg twice daily plus an appropriate background regimen. QoL was measured by the Functional Assessment of HIV Infection (FAHI) questionnaire. *Results*. 67% women and 77% men, including 67.4% black, 76.0% Hispanic, and 73.8% white patients, completed the trial. Baseline total FAHI scores were similar between sexes and races. Total FAHI of the entire population improved by Week 4 (*P* < .05); near-maximum changes obtained by Week 12 were maintained through Week 48. Women and black patients demonstrated larger improvements in total FAHI versus men, and Hispanic and white patients, respectively. *Conclusion*. HRQoL improved in all sex and racial/ethnic groups. Sex-based and race-based differences in improvements in FAHI subscales may provide insight into subtle differences of HIV-1 and treatment on HRQoL in different populations.

## 1. Introduction

Improvements in antiretroviral (ARV) therapy have increased the life expectancy of patients with HIV-1 infection, and HIV is now considered a manageable chronic disease [1]. The maintenance of a high quality of life (QoL), as well as optimization of physical, emotional, and social health, is an important aspect of disease management for HIV-1–infected patients. Health-related quality of life (HRQoL) measures are used to quantify the physical and mental aspects of HIV-1 infection that can impact a patient's overall well-being. Additionally, several studies have demonstrated a correlation between HRQoL and survival of HIV-1–infected patients [2–4], highlighting the need for monitoring and optimizing HRQoL. The GRACE (Gender, Race, And Clinical Experience) study was designed to investigate sex-based and race-based differences in outcomes with darunavir/ritonavir-(DRV/r-) based therapy in treatment-experienced patients. The primary outcomes of the GRACE study have been reported [5]. Here, we report the HRQoL results from GRACE by sex and race over 48 weeks.

## 2. Methods

### 2.1. Study Design and Patients

GRACE was a 48-week, open-label, Phase IIIb study conducted at 65 study sites across the United States, Canada, and Puerto Rico. Treatment-experienced adults (HIV-1 RNA ≥ 1000 copies/mL) received DRV/r 600/100 mg twice daily plus an appropriate ARV background regimen, chosen on the basis of resistance testing [5]. Details of the study design and the primary outcomes of the GRACE study have been reported elsewhere [5]. Human experimentation guidelines of the US Department of Health and Human Services were followed in the conduct of this clinical research, the research protocol was reviewed and approved by institutional review boards for all 65 study sites, and all participants provided written informed consent.

### 2.2. Study Evaluations

Health-related QoL was measured by the Functional Assessment of HIV Infection (FAHI) questionnaire, which contained 47 questions measuring five functional subscales [6, 7]: physical (PWB), emotional (EWB), functional and global (FGWB), and social (SWB) well-being, as well as cognitive functioning (CF). The range of possible scores for each subscale was as follows: PWB and EWB, 0 to 40; FGWB, 0 to 52; SWB, 0 to 32; CF, 0 to 12. The total FAHI score, with possible scores ranging from 0 to 176, was calculated as the sum of all five subscale values, with higher scores indicating better results. Each subject completed the FAHI questionnaire at the beginning of the study visit, before other assessments, at study entry, and at weeks 4, 12, 24, and 48 or at early withdrawal. Questionnaires completed at early withdrawal visits were not included in the analyses. The time recall period was the previous 7 days. Post hoc analyses were conducted in order to investigate factors that were associated with improvement in total FAHI score over 48 weeks. 

### 2.3. Statistical Analyses

Analyses were performed on the observed population. Imputed values were derived for each of the five subscales of the FAHI questionnaire. Imputed values were calculated if at least 50% of the items in that subscale were present using the mean of the other items in the subscale, rounded to the nearest integer; if greater than 50% of the items in a given subscale were missing, no imputation was calculated. Results from intention-to-treat–last-observation-carried-forward analyses were similar to results obtained from the observed population and are not presented here. 

In the post hoc analysis that investigated factors associated with improvement in total FAHI score, 18 covariates were evaluated using a repeated measure analysis; covariates significant at the *P* < .10 level were considered for the multivariate analysis. The multivariate analysis was performed using a forward stepwise selection with stay criteria of *P* < .05. If two or more covariates selected from the univariate analyses were highly correlated, only the most significant was considered for inclusion in the multivariate models to avoid issues with collinearity.

## 3. Results

### 3.1. Patient Population and Baseline Characteristics

The GRACE study enrolled 429 patients, of whom 66.9% were women, 61.5% were black, 22.4% were Hispanic, and 15.2% were white. In the intent-to-treat population, overall, 53.4% achieved virologic response (HIV RNA < 50 copies/mL; women, 50.9%; men, 58.5%; black, 48.5%; Hispanic, 61.5%; white, 60.0%) [5, 8]. In the time-to-loss of virologic response–non-virologic-failure censored analysis of the overall population that censored patients who discontinued for reasons other than virologic failure, 73.2% of patients (women, 73.0%; men, 73.5%; black, 68.8%; Hispanic, 79.7%; white, 78.0%) achieved virologic response [5, 8]. 

**Figure 1:**
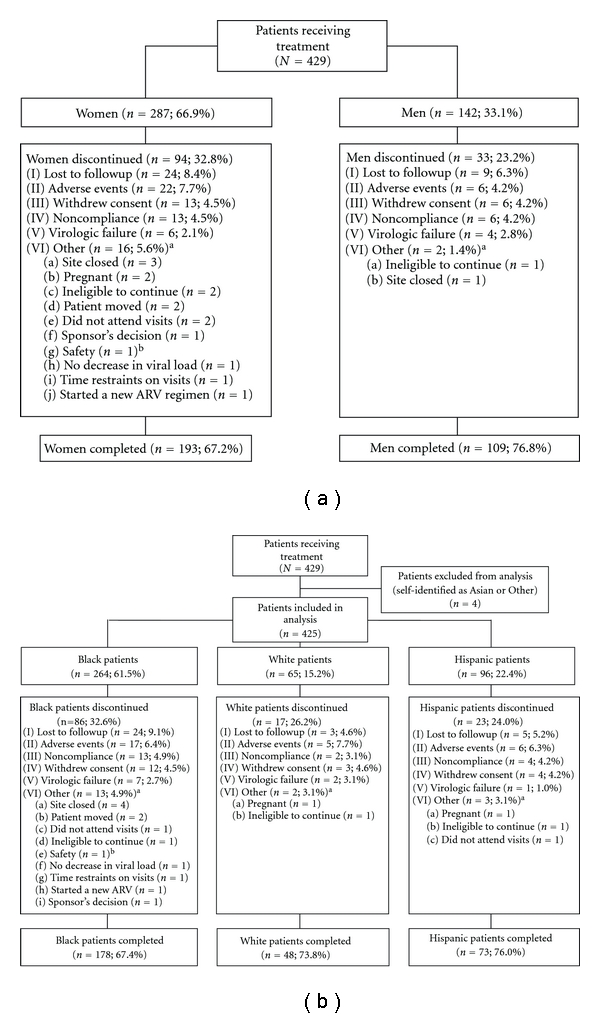
Study disposition by (a) sex and (b) race. ^a^“Other” classification was selected by the investigator as reason for discontinuation. ^b^Older patient taking too many concomitant medications; ARV: antiretroviral.

The HRQoL analysis included the 193 women (67%) and 109 men (77%) who completed the trial (Figure 1(a)). By race, 178 black (67.4%), 73 Hispanic (76.0%), and 48 white (73.8%) patients completed the trial (Figure 1(b)). Women had a higher rate of discontinuation compared with men, mostly due to reasons other than virologic failure, and black patients had a higher rate of discontinuation compared with Hispanic and white patients, mostly due to loss to followup and other reasons (Figures 1(a) and 1(b)). 

At baseline, women were younger and had a higher average body mass index than men (Table 1). Men and black patients had more advanced disease at baseline than women and Hispanic or white patients, respectively, with lower median CD4+ counts and higher proportions of US Centers for Disease Control and Prevention Class C disease (Table 1). Four patients who self-identified as Asian or other race were not included in the analyses by race due to their low numbers.

### 3.2. Health-Related Quality of Life: Total Functional Assessment of HIV Infection Score

**Figure 2:**
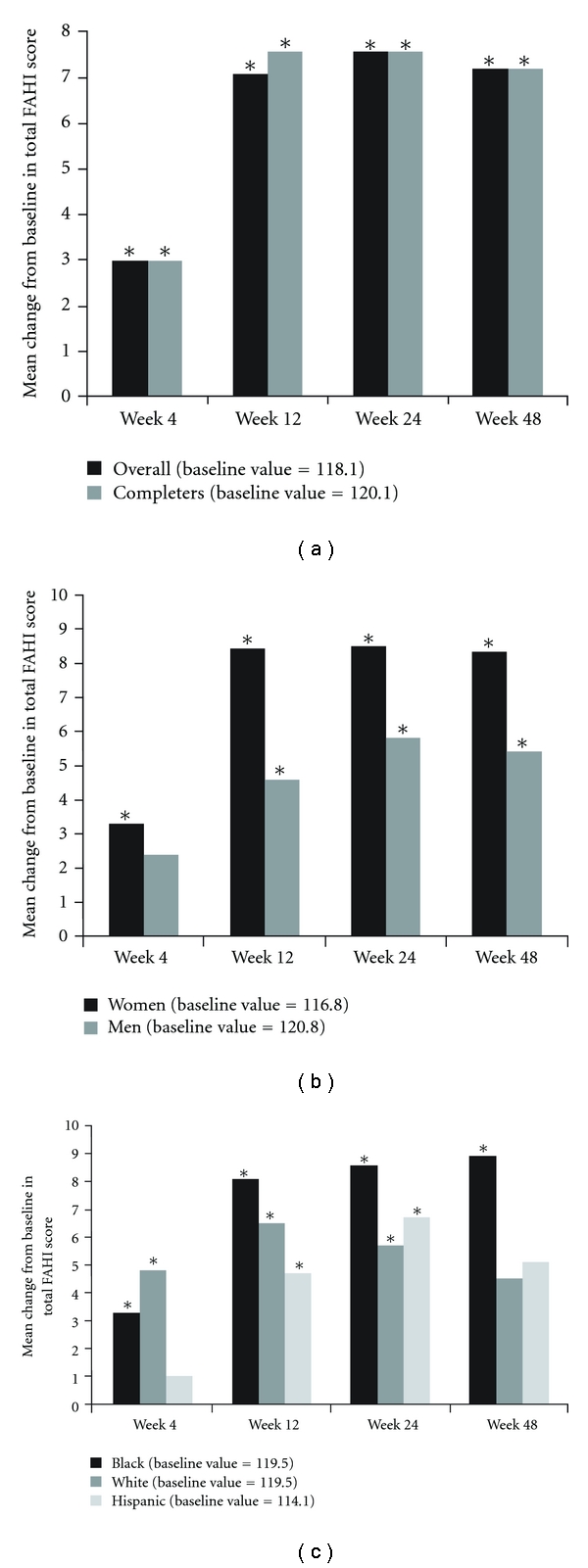
Mean change from baseline in total FAHI score in (a) the overall population and completers^a^, (b) by sex, and (c) by race^b^ (observed). *Statistically significant change from baseline (*P* < .05). ^a^Excludes patients who discontinued the trial. ^b^Four patients self-identified as Asian or other and were not included in the analysis by race due to small sample size; FAHI: functional assessment of HIV infection.

At baseline, the total FAHI scores for the overall population ranged from 26 to 176. Women had slightly lower total FAHI scores compared with men (116.8 versus 120.8), and Hispanic patients had slightly lower total scores compared with black or white patients (114.1, 119.5, and 119.5, resp.; Figure 2); neither sex-based nor race-based differences in baseline scores were statistically significant. The total FAHI score of the entire GRACE population improved significantly by Week 4 (Figure 2(a)). Near-maximum changes were achieved by Week 12 and were maintained through Week 48. Similar patterns of improvement were observed for both sexes and all races (Figures 2(b) and 2(c)). Women demonstrated larger improvements in total FAHI scores compared with men, and black patients demonstrated larger improvements compared with Hispanic and white patients. Overall, only 3% of the subscales were imputed for missing data.

The post hoc repeated measure analysis to investigate factors associated with improved FAHI over 48 weeks identified five factors with *P* < .10 (Table 2) that were considered for the multivariate analysis. The post hoc multivariate analysis subsequently identified lower baseline FAHI score, lower baseline CD4+ cell count, confirmed virologic response, and analysis time point as significantly associated with improvement in FAHI score over 48 weeks (Table 3). Notably, neither sex nor race was found to be significant in the final model, and there was no interaction between the two variables. 

In a post hoc sensitivity analysis, it was noted that patients with lower baseline FAHI scores were significantly more likely to discontinue (*P* = .044) than patients with higher baseline HRQoL scores. To assess the impact of discontinuations, an additional post hoc sensitivity analysis evaluated only patients who completed the trial. When patients who discontinued the trial were excluded and only patients who completed the trial were analyzed, the total FAHI score still improved from baseline to Week 48 (Figure 2(a)) to a similar extent as in the total population. 

### 3.3. Health-Related Quality of Life: Functional Assessment of HIV Infection Subscales

The EWB and PWB subscales showed significant changes from baseline to Week 12, which were maintained through Week 48 for the overall population and across all sex and race subgroups (Table 4). Women and black patients demonstrated the largest changes in EWB compared with the other subgroups. The FGWB and SWB subscales showed small improvements from baseline through Week 48 overall. Changes in the FGWB subscale were only significant for women and black patients (Table 4). The CF subscale values did not change over 48 weeks for the overall population, or for any of the sex and race subgroups. 

## 4. Discussion

Many factors can impact the HRQoL of patients with HIV-1, including the tolerability of different ARV agents. Differences in safety/tolerability, pharmacokinetic parameters, and/or efficacy of ARVs could potentially contribute to sex-based or race-based differences in HRQoL scores. To date, studies investigating sex-based and race-based differences in HRQoL for HIV-1–infected patients have reported conflicting results [9–12]. In the GRACE study, HRQoL significantly improved for all participants. Near-maximal improvements were reached by Week 12 and were maintained through Week 48. When only patients who completed the trial were analyzed, similar improvements in total FAHI score over time were still seen, suggesting that the improvements in the overall population were not an artifact of patients with low HRQoL scores discontinuing the trial.

All sex and racial/ethnic groups demonstrated improvements in HRQoL over 48 weeks. These results are consistent with those from a previous study of 1178 patients receiving ARV therapy, which also showed that HRQoL improved over time, regardless of sex [11]. In univariate assessments, the largest improvements in total FAHI scores were seen in women and black patients, despite the fact that these two groups both had lower virologic response rates and higher discontinuation rates compared with men and with Hispanic and white patients, respectively. The differences seen here in total FAHI scores were driven by the larger increases in the EWB and FGWB subscales for women and black patients compared with the other subgroups. The post hoc multivariate analysis, however, found that neither black race nor sex was significantly associated with improved total FAHI scores over 48 weeks. Given that sex and race were not associated with improved total FAHI in the multivariate analysis, it is likely that differences in the change in HRQoL by sex and race were at least partially driven by differences in baseline characteristics, such as disease severity. These results are in agreement with those from another study of 287 HIV-1–infected women who completed a HRQoL assessment during their current treatment regimen. This study also found that race had no bearing on HRQoL [10]; however, changes in HRQoL over time were not assessed.

Lower baseline FAHI scores, lower baseline CD4+ cell counts, and confirmed virologic response were identified as significantly associated with improved total FAHI scores over 48 weeks. CD4+ cell count and general disease state have previously been associated with absolute HRQoL scores in other studies [10, 12, 13]. Higher CD4+ cell counts have been linked to higher HRQoL scores in women and men [10, 13], whereas the presence of AIDS has been linked to lower self-perceived physical health scores in both sexes [12]. The DUET study, which investigated the efficacy and safety of etravirine in treatment-experienced patients, also demonstrated an association between baseline FAHI score and CD4+ cell counts and improvement in FAHI scores over 24 weeks [14]. 

Results from this study suggest that DRV/r-based, optimized, ARV therapy over 48 weeks results in significant improvement in self-perceived HRQoL, regardless of sex and race. However, due to the single-arm design of this study, we cannot conclude whether this improvement is better or worse than those associated with other ARV regimens. Previous studies of sex-based and race-based differences in HRQoL scores have demonstrated conflicting results, with some studies noting differences in overall HRQoL between the sexes or races and other studies reporting no differences [9–12]. These studies, however, are limited due to low proportions of women or people of color and/or the enrollment of women only, with no male group for comparison. In contrast, the large proportion of women enrolled in GRACE, along with a sufficient number of men and the large proportion of people of color, may allow more clinically meaningful conclusions to be drawn regarding sex-based and race-based differences in HRQoL. 

Although the overall conclusions from previous studies about sex-based or race-based differences in HRQoL are contradictory, previous studies do suggest that factors affecting HRQoL scores may vary between sexes. One study showed that longer duration of infection and low levels of social support were linked to lower HRQoL scores in women, whereas difficulty taking ARV tablets, intravenous drug use, and low levels of social support were associated with lower HRQoL scores in men [12]. These sex-associated differences may make it possible to tailor HIV treatments and treatment support to women and men, thus maximizing their overall QoL.

In this trial, confirmed virologic response was found to be significantly associated with improved total FAHI scores. In the GRACE trial, women and black patients had lower response rates and higher discontinuation rates than the other sex and race subgroups [5]. Based on this, lower improvements in FAHI scores in these two groups may have been expected. However, the opposite was observed, with women and black patients demonstrating the largest improvements in FAHI scores over 48 weeks. These contrary results suggest the need to measure alternative outcomes in clinical trials, as virologic response alone may not be wholly indicative of a patient's overall improvement while on ARV therapy. The GRACE study was designed with patient support in mind, with opportunities to apply for sponsorship for site-specific recruitment and retention activities [15]. These activities may have contributed to the subjects' comfort level with both treatment and study participation and may have led to a greater sense of attachment to site staff and care delivery. It is likely that factors distinct from virologic response may be influencing patients' overall HRQoL, particularly for women and black patients, and these should be investigated in the future. 

Interestingly, we found that patients with lower baseline FAHI scores were significantly more likely to discontinue than patients with higher baseline HRQoL scores. Although data are lacking with regard to the association between HRQoL and retention of patients in clinical trials, other studies have linked lower HRQoL scores to decreased survival of HIV-1–infected subjects [2–4]. Furthermore, measures of HRQoL in a large veteran outpatient population have also been shown to predict survival more generally [16]. In the future, it may be possible to identify patients with a higher risk of discontinuation based on their baseline HRQoL scores; these patients could then be more closely monitored, potentially improving retention in care. This approach deserves further study. 

## 5. Conclusions

The sex-based and race-based differences in the improvements in total FAHI score and FAHI subscales may provide insight into the subtly different effects of HIV-1 infection and treatment on HRQoL in different populations. The data from GRACE on HRQoL by sex and race may provide valuable information into methods resulting in future optimization of treatments and retention in care for specific populations of HIV-infected patients. 

## Figures and Tables

**Table 1:** Baseline demographics and disease characteristics by sex and race^a^.

Parameter	Women *n* = 287	Men *n* = 142	Black *n* = 264	Hispanic *n* = 96	White *n* = 65
Age, mean (SE), years	41.7 (0.63)	45.2 (0.75)	43.0 (0.62)	40.3 (1.05)	45.5 (1.13)
Gender, *n* (%)					
Female	287 (100)	—	191 (72.3)	60 (62.5)	34 (52.3)
Male	—	142 (100)	73 (27.7)	36 (37.5)	31 (47.7)
BMI, mean (SE), kg/m^2^	28.2 (0.44)	25.4 (0.42)	27.6 (0.44)	26.8 (0.58)	26.7 (0.88)
Duration of infection, mean (SE), years	10.9 (0.32)	12.2 (0.49)	11.0 (0.34)	10.5 (0.58)	13.8 (0.66)
Viral load, mean (SE), log_10_ copies/mL	4.65 (0.05)	4.73 (0.07)	4.66 (0.06)	4.68 (0.09)	4.73 (0.10)
CD4+ count, median (range), cells/mm^3^	210 (1, 868)	175 (2, 1125)	179 (1, 868)	208 (1, 1125)	249 (6, 826)
CDC Class C, *n* (%)	102 (35.5)	67 (47.2)	111 (42.0)	33 (34.4)	22 (33.8)
Prior use of ≥2 PIs, *n* (%)	168 (58.5)	92 (64.8)	156 (59.1)	60 (62.5)	42 (64.6)
PSS of the OBR^b^, mean (SD)	2.0 (0.65)	2.0 (0.81)	2.0 (0.72)	1.9 (0.67)	2.0 (0.67)
Hepatitis B surface antigen (positive), *n* (%)	12 (4.2)	7 (4.9)	15 (5.7)	3 (3.1)	1 (1.5)
Hepatitis C antibody (positive), *n* (%)	39 (13.6)	25 (17.6)	41 (15.5)	12 (12.5)	11 (16.9)

^a^Two women and two men self-identified as Asian or other and were not included in the analysis by race. ^b^By virco TYPE; SE: standard error; BMI: body mass index; CDC: Centers for Disease Control and Prevention; PI: protease inhibitor; PSS: phenotypic susceptibility score; OBR: optimized background regimen; SD: standard deviation.

**Table 2:** Results from the univariate analysis to investigate factors associated with improvements in total FAHI score over 48 weeks.

Covariate	*P* value
Baseline FAHI	<.0001
Baseline CD4+ count	.0052
Confirmed virologic response	.0335
History of psychological illness	.0570
Baseline log_10_ viral load	.0862
Stage of HIV infection	.1441
Sex	.1979
Presence of etravirine in the OBR	.2062
Alcohol use	.2700
Duration of diagnosis	.2995
Black race versus other	.3490
Incidence of grade 2–4 AE	.4374
Smoker	.5272
Presence of hepatitis C antibody	.5938
Age	.7183
Structured treatment interruption	.7757
Adherence	.8049
Drug use	.9548

FAHI: Functional Assessment of HIV Infection; OBR: optimized background regimen; AE: adverse event.

**Table 3:** Factors associated with improvements in total FAHI score over 48 weeks in the final multivariate model.

Covariate	*P* value
Lower baseline FAHI	<.0001
Confirmed virologic response	.0045
Analysis time point^a^	.0002
Lower baseline CD4+ count	.0077

^a^Total FAHI score increases over time; FAHI: Functional Assessment of HIV Infection.

**Table 4:** Mean change from baseline in FAHI questionnaire subscales (observed^a^).

	*n*	Overall	*n*	Women	*n*	Men	*n*	Black	*n*	Hispanic	*n*	White
PWB												
BL	424	29.4	283	28.8	141	30.6	262	29.5	94	29.7	64	29.0
Wk 12Δ	359	2.6^b^	236	2.7^b^	123	2.3^b^	218	2.6^b^	82	2.6^b^	55	2.2^b^
Wk 48Δ	298	2.2^b^	190	2.3^b^	108	2.0^b^	176	2.3^b^	71	2.1^b^	48	1.8^b^

EWB												
BL	423	25.1	283	24.5	140	26.5	261	25.4	94	23.3	64	26.7
Wk 12Δ	358	3.1^b^	235	3.6^b^	123	2.2^b^	218	3.6^b^	82	2.1^b^	54	2.8^b^
Wk 48Δ	297	3.8^b^	190	4.5^b^	107	2.5^b^	175	4.1^b^	71	3.8^b^	48	2.3^b^

SWB												
BL	423	20.5	283	20.7	140	19.9	261	21.0	94	18.5	64	21.3
Wk 12Δ	359	0.2	236	0.4	123	−0.2	218	0.2	82	−0.5	55	0.9
Wk 48Δ	296	0.5	189	0.3	107	1.0	174	0.8	71	0.1	48	0.6

CF												
BL	423	8.2	283	8.0	140	8.7	261	8.3	94	8.0	64	8.2
Wk 12Δ	359	0.1	236	0.3	123	−0.2	218	0.2	82	−0.1	55	0.1
Wk 48Δ	296	−0.1	189	0.1	107	−0.4	174	0.0	71	−0.3	48	−0.2

FGWB												
BL	423	34.9	283	34.8	140	35.2	261	35.4	94	34.5	64	34.3
Wk 12Δ	359	1.1^b^	236	1.4^b^	123	0.6	218	1.5^b^	82	0.6	55	0.5
Wk 48Δ	297	0.7	190	1.0	107	0.3	175	1.5^b^	71	−0.6	48	0.1

^a^The *n* value varies for each parameter. ^b^Significant change (*P* < .05); FAHI: Functional Assessment of HIV Infection; PWB: physical well-being; BL: baseline; Wk: week; Δ: change from baseline; EWB: emotional well-being; SWB: social well-being; CF: cognitive functioning; FGWB: functional and global well-being.
